# A Global Review of the Impacts of the Coronavirus (COVID-19) Pandemic on Radiology Practice, Finances, and Operations

**DOI:** 10.3390/life13040962

**Published:** 2023-04-06

**Authors:** Kishan Patel, Arnav Rashid, Luke Spear, Ali Gholamrezanezhad

**Affiliations:** 1 Keck School of Medicine, University of Southern California, Los Angeles, CA 90033, USA; kishannp@usc.edu; 2 Department of Biological Sciences, Dana and David Dornsife College of Letters, Arts, and Sciences, University of Southern California, Los Angeles, CA 90089, USA; 3 Department of Radiology, Keck School of Medicine, University of Southern California, Los Angeles, CA 90033, USA

**Keywords:** computed tomography (CT), coronavirus, COVID-19, hospital, imaging volumes, MRI, pandemic, radiology department, Relative Value Units (RVUs), clinical operations

## Abstract

The Coronavirus Disease 2019 (COVID-19) pandemic ushered in rapid changes in healthcare, including radiology, globally. This review discusses the impact of the pandemic on various radiology departments globally. We analyze the implications of the COVID-19 pandemic on the imaging volumes, finances, and clinical operations of radiology departments in 2020. Studies from health systems and outpatient imaging centers were analyzed, and the activity throughout 2020 was compared to the pre-pandemic activity, including activity during similar timeframes in 2019. Imaging volumes across modalities, including MRI and CT scans, were compared, as were the Relative Value Units (RVUs) for imaging finances. Furthermore, we compared clinical operations, including staffing and sanitation procedures. We found that imaging volumes in private practices and academic centers decreased globally. The decreases in volume could be attributed to delayed patient screenings, as well as the implementation of protocols, such as the deep cleaning of equipment between patients. Revenues from imaging also decreased globally, with many institutions noting a substantial decline in RVUs and revenue compared with pre-COVID-19 levels. Our analysis thus found significant changes in the volumes, finances, and operations of radiology departments due to the COVID-19 pandemic.

## 1. Introduction

The rapid onset of the novel Coronavirus Disease 2019 (COVID-19) outbreak created a global challenge to worldwide operations, logistics, and finances. Hospital patient volumes decreased during the beginning stages of the pandemic in multiple medical disciplines, including radiology, emergency, and surgery departments [1,2,3,4]. Decreases in volumes were also seen in individual practices, such as those in dermatology and rheumatology [5,6]. The effects on patient volumes during the COVID-19 pandemic were also seen internationally, with hospitals around the world noting a decrease in hospital revenue [7,8]. The American Hospital Association estimated that between March and June 2020, American hospitals lost on average $50.7 billion per month [8]. The decrease in overall patient volumes led to a decreased need for imaging within hospital systems. Consequently, both imaging volumes and revenue generation were lower during the initial stages of the COVID-19 pandemic [9,10].

During the COVID-19 pandemic, certain radiology divisions were affected more heavily than others, as illustrated by differences in imaging volume decreases [11]. Additionally, the reduction in imaging volume led to changes in radiology department and outpatient imaging center operations. Such operational changes included utilization of personnel, such as radiology staff, as well as resources, such as portable imaging devices. The effects of such operational changes also led to alterations in teaching at academic centers and created changes that may persist at the end of the pandemic. The goal of this review is to synthesize the effects of the COVID-19 pandemic to imaging volume, radiology staffing, patient screening, and imaging workflow to both public and private hospitals within and outside the United States. This review will also discuss the financial implications of the COVID-19 pandemic to both hospitals and outpatient centers.

## 2. Materials and Methods

### 2.1. Search Criteria

The current literature review was performed on the PubMed and Google Scholar databases between the dates 20 November 2022 and 27 December 2022. The search criteria included the following individual terms: “COVID-19”, “pandemic”, “operations”, “staffing”, “revenue”, “costs”, “volume”, “census”, “radiology”, “department”, “hospital”, “outpatient”, “clinic”, “medical center”, “United States”, “public hospital”, “private hospital”, “workflow”, “imaging”, “finance”, “economics”, “RVU”, “salary”, “income”, “government”, “protocol”, “guideline”, “relief”, “funding”, and “productivity”. The above terms were searched in multiple combinations. While published literature on the effects of the COVID-19 pandemic on every country is not available, searches were conducted to obtain results on both public and private radiology practices worldwide.

### 2.2. Selection Criteria

From the search criteria, articles that discussed the impact of the COVID-19 pandemic to either a hospital, medical center, or specific department or division were selected for initial review. Articles describing the effect on outpatient centers or private practice radiology practices were also selected. The abstracts and manuscript texts were analyzed for their relevance to the goals of the current review article. The most relevant articles were selected based on the presence of statistical measures and the size of the study. Articles from both hospitals and clinics within and outside of the United States were included in the final review.

### 2.3. Analysis

Analysis of the selected articles was performed to measure the average effects of the COVID-19 pandemic to the operations and finances of the hospital or outpatient setting. Changes in imaging volume were determined by either gross changes in the number of imaging exams performed or changes in the percentage of imaging exams performed. Changes in finances were determined from the provided revenue totals or the changes in Relative Value Units (RVUs) or Work Relative Value Units (wRVUs). Analysis of operations was based on both anecdotal evidence presented within each reviewed article and quantitative measures as statistically found by the authors of these articles. Unless otherwise noted, “pre-pandemic” refers to prior to the year 2020. When comparing changes in volume or revenue, values are compared between the same time periods in 2020 and 2019, unless otherwise stated.

## 3. Results

### 3.1. Volume

**Figure 1:**
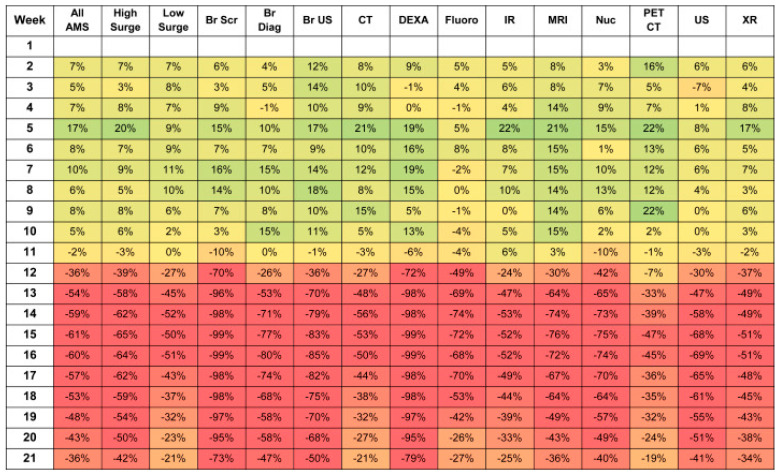
Percent Changes in Weekly Imaging Volume in the First 21 Weeks of 2020 Compared with 2019 at Six Academic Medical Centers. Green = Increase in number of radiological studies; Red = Decrease in number of radiological studies; Yellow = No change in number of radiological studies.

Imaging volumes were noted to be decreased at all hospitals and outpatient centers found in this review study. A 525-bed public hospital and level I trauma center in Denver, Colorado, noted overall imaging volumes to be 55% of pre-COVID-19 imaging volumes between the months of 27 March 2020 and 23 April 2020 [9]. Volumes of both chest imaging and deep venous thrombosis (DVT) studies were decreased during this period. Imaging volumes increased after the initial wave to levels similar to the pre-COVID-19 pandemic levels [9]. A study of six academic medical centers in California, New York, Michigan, and Massachusetts found that imaging volumes decreased most heavily between 9 March 2020 and 20 April 2020. The range of volume decrease ranged from 40% to 70%, with academic centers located in high-surge areas seeing an increased drop in volume [12]. Overall, the largest drops in volume occurred in the breast and DEXA subsections, with breast volume dropping 99% at one center. In low-surge areas, positron emission tomography/computed tomography (PET/CT) was the modality with the lowest drop in comparison to plain film x-ray and interventional radiology being the lowest drop in high-surge areas (Figure 1) [12]. Similarly, a large level-one trauma center and safety net hospital in Los Angeles noted a 25% decrease in volume in March 2020 and a 45% drop in volume in April 2020 for all inpatient studies [13].

At the largest health system in New York, an 88% drop in breast imaging volume was seen across all modalities. There was a 64% decrease in ultrasound, 94% in mammography, and 74% in MR imaging studies. Additionally, mammography volumes decreased by as much as 99% during the trough of the study volume [14].

**Figure 2:**
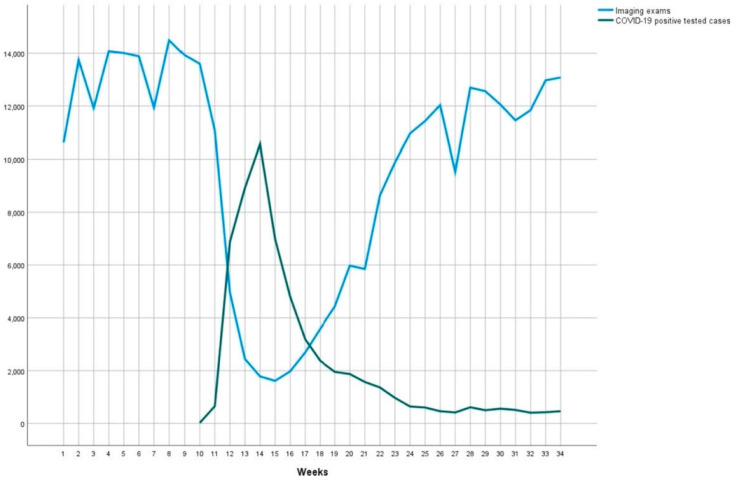
2020 Imaging Utilization vs. COVID-19 Positive Cases.

Outpatient imaging centers also witnessed a decrease in overall imaging volumes. A retrospective analysis of a large integrated healthcare system in the New York City metropolitan region noted a significant decrease in imaging volumes in 2020 compared with 2019 [15]. From 1 January 2020 to 1 March 2020, imaging volumes increased by 7.6%; from 2 March 2020 to 12 April 2020, volumes decreased by 56.6%; and from 13 April 2020 to 23 August 2020, volumes decreased by 30.1% (Figure 2) [15]. At an outpatient center at a pediatric hospital in London, there was a decrease in the overall volume from 67,806 records in the pre-COVID-19 period to 48,250 in the COVID-19 period [16].

Additionally, private radiology practices may have seen imaging volumes decrease by up to 80%. A case study of one practice in Michigan noted a 55.4% decrease in imaging volume from the periods of 6 January 2020 to 22 March 2020 and 23 March 2020 to 3 May 2020 [17].

A study of a large hospital system in Saudi Arabia noted a decrease in overall imaging volumes between 2019 and 2020. Decreases in imaging volumes were seen mostly between the months of April and September 2020, where there was a greater than 40% reduction in imaging volumes compared with 2019 [18]. Outpatient imaging was the most affected followed by inpatient imaging volumes. Ultrasound, mammography, and magnetic resonance imaging (MRI) were the modalities most affected during this period [18]. A study of imaging volume across Victoria, Australia, noted a 20.7% decrease in imaging volume between 11 March 2020 and 21 April 2020 compared with the same timeframe in 2019 [10]. The subsections of nuclear medicine, mammography, and magnetic resonance imaging had the largest decreases in volume [10]. A multicenter survey of chest CT scans in Iran between 2019 and 2020 found that there was a 50.2% increase in chest CT volume compared with 2018 to 2019. A decrease of 123.9%, 10.8%, and 4.3% was seen for MRI, brain CT, and abdominopelvic CT imaging, respectively [19].

### 3.2. Finances

**Figure 3:**
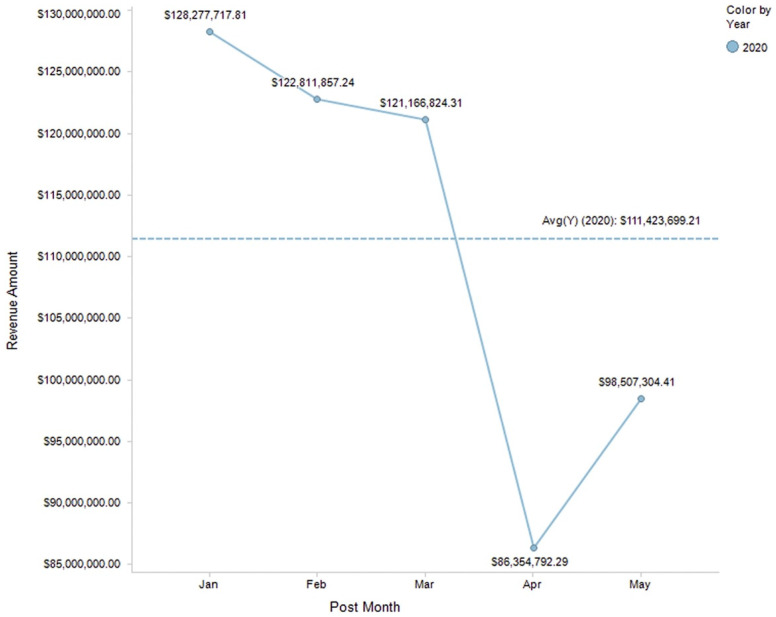
2020 Monthly Gross Radiology Charges at Washington-based Medical Center.

Revenue from imaging finances was seen to decrease globally during the COVID-19 pandemic. A study performed within the University of Washington radiology department found that, between January 2020 and May 2020, there was a decline in monthly imaging revenue, with the largest decline in the month of April, where imaging revenue was $86,354,792 compared with $121,166,824.21 in March (Figure 3) [20]. A retrospective study by a large New York health system noted that imaging volume fell by 57% during the surge period and average work RVUs fell by 69% compared with pre-COVID-19 volumes and work RVUs [21]. In this study, the surge period was defined as the period between 2 March 2020 and 10 May 2020. The median RVU per study decreased from 0.76 in the pre-surge period to 0.31 during the surge period [21].

A multi-subspecialty radiology practice in Michigan noted an overall average decrease in wRVUs of 52.1% between the intra-pandemic weeks of 2020, defined as 16 March 2020 to 18 May 2020, and the same time frame in 2019 [22]. The largest decreases in wRVUs were seen with mammography, MRI, and non-PET nuclear medicine modalities, which had decreases of 81.6%, 69.7%, and 70.7%, respectively. The departments with the largest wRVU decreases were breast, neuro, and musculoskeletal, with decreases of 75.1%, 70.6%, and 66.0%, respectively [22].

A study of the Indian health system predicted that the revenues of both inpatient and diagnostic centers had decreased between 60 and 70% during the lockdown period of the pandemic [23]. The largest decreases in revenue were seen in ultrasound, DEXA, and mammography imaging [23]. A study of the largest hospital in Cameroon found a 35.83% decrease in revenue in 2020 compared with 2019, with the largest revenue decrease of 40.31% occurring in April 2020 [7].

### 3.3. Operations

Clinical operations within hospitals and group practices were affected during the COVID-19 pandemic. A survey of radiology practices in the United States noted that 31.1% of hospitals or group practices reassigned a number of radiologists to paid time off (PTO) [24]. A study from a large academic center in Massachusetts found that between February and April 2020, 24.9% of radiology staff were reassigned to roles outside of their typical role [25]. Faculty and training physicians were reassigned to roles, including employee health attestation and PPE distribution, virtual radiology consultants on rounds, and medical staff on COVID-19 floors. Auxiliary staff, such as radiology technicians, were reassigned to a COVID-19 field hospital and to PPE distribution. Radiology faculty from other departments were also asked if they may read in the thoracic and emergency radiology divisions [25].

An academic center in Turkey noted that during the pandemic, CT technologists were placed in cohorts, with each cohort being on for one week with the following next two weeks off to decrease the risk of COVID-19 transmission between staff [26].

As radiology centers recovered imaging volume after the initial pandemic waves, academic centers implemented a number of operational changes. From a survey of academic radiology centers in the United States, 87% of centers began testing patients prior to arrival at the facility, 58% created a telephone screening questionnaire, and 84% required COVID-19 testing for aerosolizing procedures [11]. The same survey also noted that 71% of backlogged studies were prioritized based on their time sensitivity [11,27].

For confirmed or suspected COVID-19 positive patients undergoing a CT or MRI scan, radiology centers followed protocols for decontamination. At a Los Angeles safety-net hospital, there was an average downtime of 1 h for aeration and cleaning of the CT scanner in the case of preparing the room for a COVID-19 negative patient after the imaging of a COVID-19 positive patient occurred. Similarly, an average downtime of 1.25 h was seen for the MRI scanner [13]. Additionally, staffing in the radiology department was decreased with residents decreasing onsite hours from 50 to 20 h per work week. Furthermore, radiology department healthcare workers who had high-risk encounters with patients or who had a possible exposure to respiratory secretion droplets would be required to stay at home and self-monitor for COVID-19 symptoms.

The use of mobile imaging units were seen in some institutions, with one Australian institution noting a 1.7-fold increase in portable x-ray use [28]. Similarly, a Singaporean hospital noted an increase in portable x-ray use for both standard chest x-rays, as well as for more advanced plain film studies [29]. At a hospital in Jaipur, India, portable x-rays were often utilized for COVID-19 diagnosis in lieu of CT imaging to reduce overall CT imaging burden [30].

There was an increase in the number of remote readings during the pandemic. A hospital-based radiology department in Houston shifted from having 100% onsite staff to 80% reading offsite using home PACS workstations that were delivered within a two-week period in March 2020 [31].

## 4. Discussion

The COVID-19 pandemic affected radiology departments in both the academic and private settings worldwide. The effects of the pandemic led to overall decreases in imaging volume and resultant imaging revenue. Hospital systems and clinics often resorted to altering their operational workflow to accommodate such changes. These changes were seen as alterations in staffing, healthcare delivery, study prioritization, and COVID-19 symptom screening. Organizational committees, such as the American College of Radiology, issued recommendations for the use of certain imaging throughout the pandemic [32]. Additionally, while some radiology departments implemented protocols associated with the entire hospital system, others created department-specific protocols [13,33,34,35,36,37].

This review focuses on the effect of the COVID-19 pandemic during the first lockdown period. The initial lockdown period, if initiated, varied by country [38]. The majority of lockdown periods occurred near the month of March 2020 [39]. The effect of the lockdown period on hospital admissions was similar around the globe [40,41]. Many hospital systems noted a dramatic decrease in total emergency room visits and inpatient admissions during the lockdown period. For countries following a tiered lockdown approach, the largest delta decrease in admissions was seen during the strictest lockdown period [42]. Consequently, at these hospital centers, imaging volumes dropped to a fraction of pre-COVID-19 volumes.

Volume decreases were seen both nationally and internationally in both private practices and academic centers. From our review, decreases in inpatient volume were much less than decreases seen in the same hospital’s outpatient centers. On the inpatient side, decreases varied per institution. While some institutions noted an overall decrease in chest imaging volume, others noted a substantial increase [9,19]. This discrepancy may be explained by differences in surge areas and local protocols. Furthermore, outside of the United States, chest imaging was heavily utilized to diagnose the COVID-19 disease prior to the availability of COVID-19 nasal kits. Examples of such a diagnostic approach were seen in countries such as Iran, China, and South Korea [38]. This may have led to an increase in overall chest imaging volumes during the pandemic in these countries. Many hospitals also followed a deep cleaning protocol after the imaging of a patient with a known COVID-19 infection [20,43]. This led to downtimes of up to 1 h in many facilities, resulting in an equipment bottleneck and subsequent decrease in imaging volume [13,44]. Furthermore, many hospitals in the United States instituted guidelines for COVID-19 imaging based on pre-test probability, which decreased the total number of patients receiving chest imaging, in contrast to what may have been seen internationally [32]. Additionally, as overall hospital volumes decreased for non-COVID-19 related causes, total imaging requirements decreased [45].

Changes in imaging volume per modality and department also varied significantly between institutions. Some institutions noted a decrease in imaging volume in the musculoskeletal departments, while others noted decreases in the imaging volume of the nuclear medicine departments. However, decreases were most consistently seen in the breast imaging department regardless of institution type. Decreases in breast imaging may have occurred secondary to guidance from organizational communities. The Canadian Society of Breast Imaging and Canadian Association of Radiologists Joint Position on COVID-19 recommended deferring screening mammography by 6–8 weeks [14]. Similarly, the Society of Breast Imaging recommended delaying screening by several weeks to a few months [14]. International committees also recommended a similar delay in screening during the initial lockdown periods of the pandemic [14]. While the ramifications of delayed screening to patient breast cancer diagnosis and care are yet to be fully determined, early studies forecast that the disruptions of routine screening during the first lockdown period will have a small long-term effect on overall breast cancer mortality [46,47]. Future strategies to decrease the number of delayed studies in a similar pandemic may involve a combination of telehealth services and mobile screening services. Telehealth services may be utilized to identify higher-risk individuals. A mobile mammography unit may limit exposure of the patient to the hospital, as well as staff to high-risk individuals compared with the traditional in-office mammography. A mobile mammography unit was successfully used in Taiwan during the pandemic. This helped to limit percentage decrease in the number of breast cancer screenings to 22.2%, while traditional in-hospital exams decreased by 37.2% [48]. Future resources should be allocated to study the effectiveness of mobile mammography units.

Lung cancer screening with a low-dose CT is generally indicated by the US Preventive Service Task Force for current smokers with a 20 pack-year smoking history or those who have quit within the past 15 years. Prior to the COVID-19 pandemic, lung cancer screening rates were already lower compared to screening rates for breast, colorectal, prostate, and cervical cancer [49]. During the COVID-19 lockdown period, many lung cancer screening programs were temporarily shut down [50,51]. While some programs created telemedicine programs to limit the number of screenings to those who were higher risk, a majority of institutions temporarily halted all operations. One study noted that once screenings resumed, there was a higher proportion of patients with suspicious lung nodules (Lung-RADS 4) [50]. Furthermore, the screening rate post-COVID-19 has been significantly lower than the screening rate prior to the pandemic at multiple institutions [50,51,52]. Guided and directed efforts should be taken by hospital centers to increase the screening rate to reach pre-pandemic levels.

While thyroid cancer is not usually screened for, diagnostic procedures of the thyroid were recommended to be delayed during the pandemic [53]. It was recommended that ultrasounds be delayed for patients with the discovery of thyroid nodules on routine exam, with the exception of those who were experiencing symptoms affecting hemodynamic stability or those with a high suspicion of cancer based on clinical history. Additionally, nuclear medicine studies, such as radioactive iodine administration, were also recommended to be delayed in patients with benign conditions. With the decrease in imaging studies, there has also been a decrease in the number of fine-needle aspiration biopsies. One multi-center study noted that there was an 80.5% decrease in thyroid cytopathology specimens during the lockdown period of the pandemic [54]. While the effect of decreased biopsies and imaging is yet to be fully studied, delayed surgical intervention during the pandemic has not been shown to increase the rate of extrathyroidal extension in the general population. However, the rate of extension did increase in high-risk patients per the American Thyroid Association classifications [55].

Delays in cancer screening and imaging decreases during the pandemic resulted in many institutions noting a more progressed initial presentation of numerous cancers in the post-pandemic period compared with the pre-pandemic period. A study of a community clinic in Brazil noted a higher prevalence of advanced-stage breast cancer (stage IV) after the pandemic onset compared with prior to the pandemic onset [56]. Additionally, the early-stage breast cancer incidence was higher in the pre-pandemic period compared with after the pandemic onset [56]. Similar results were seen at a University Hospital in Egypt, where the prevalence of stage III and IV breast cancers were 27.9% higher in the post-COVID-19 pandemic group compared with the pre-COVID-19 pandemic group [57]. The proportion of T3 and T4 size tumors were also more prevalent in the post-COVID-19 pandemic group. These patients also had a 33.8% increase in the frequency of neoadjuvant therapy compared with the pre-COVID-19 group [57]. A study of head and neck cancers from the University of Arkansas found more patients diagnosed with T3- or T4-stage tumors after the pandemic onset compared with those before [58]. As it is still early in the post-COVID-19 period, the number of studies comparing cancer staging before and after the pandemic is not extensive. However, these studies demonstrate that the delayed diagnoses that have occurred during the pandemic may result in increased findings of advanced cancer stages in the upcoming years. Overall, there was a decrease in neo-adjuvant therapies during the pandemic with an increase in oral adjuvant therapies [59]. During the post-lockdown phase as imaging studies increased and larger-sized and advanced tumors were detected, more aggressive care and neo-adjuvant therapies were utilized. The long-term effect on patient mortality is still to be determined.

The decreases in total imaging volume are inherently linked to decreases in total revenue generation in both private practice and hospital-based radiology practices. Moreover, while imaging volume decreased, wRVUs may have decreased to a greater extent than volume, indicating a substantial decrease in the total payment to radiology practices [21]. RVUs are used as metrics to measure the complexity and amount of work required to perform a procedure. While there is not necessarily a correlation between time-to-read and RVU, there is a correlation between modality and RVUs [60]. Generally, MR imaging produces a higher RVU amount than imaging of the same body part on a CT, which provides a higher RVU amount than imaging of the same body part on a plain film [61]. Due to the decrease in total inpatient admissions and further decreases in advanced modalities, such as MRIs, a larger percent decrease in RVUs than percent decrease in imaging volumes was seen. A similar trend may have been seen in the outpatient sector as well but potentially due to a differing etiology. In the outpatient setting, delays were seen in many imaging modalities; however, the largest decreases were seen in the modalities of mammography, MRI, and CT according to one large academic center with over 12,000 imaging delays [11]. These modalities produce higher average RVUs compared to less delayed imaging modalities, such as plain films and ultrasound. Consequently, average reductions in radiology group practices were approximately 50% during the early stages of the pandemic. As a result, nearly 70% of respondents to a survey of 228 practices reported applying for governmental financial relief [24].

Globally, there is a relationship between average receipt value for imaging studies and average radiologist salary [62]. This relationship is magnified in the United States where radiologist compensation is often linked to productivity measured in RVUs [63]. The effect of decreased RVUs was seen in radiologist compensation in group and individual practices. One survey noted up to 50% of practices decreased staff radiologist salaries as well as bonuses, financial incentives, and retirement allocations, in addition to decreasing overall workload and hours worked [24]. Even well-funded radiology groups owned by private equity groups were told that a favorable return-on-investment was more important than the return of human capital in the post-pandemic period [64]. The long-term effects of decreased compensation are unknown. Since the beginning of the pandemic lockdown period in 2020, there has not been a sustained period of decreased global imaging volume [65]. This decrease occurs in the setting of a long-term trend in an increasing number of average imaging examinations per patient [66]. This decrease in volume, despite an increasing trend in the average number of imaging examinations, may lead to a potential labor shortage within radiology as COVID-19 protocols and guidelines regarding imaging are relaxed. Compounding this effect is the number of radiologists who are closer to the retirement age. The current radiologist labor pool in the United States is skewed right, with over 80% of currently practicing radiologists being over the age of 45 and 53% being older than 55 years [67]. Since 2004, there has been a widening gap between the demand for radiologists and the supply of radiologists, as studied by the research market firm Frost & Sullivan [68]. We expect that this trend may be exacerbated in the setting of (1) an increasing of number of retiring radiologists and (2) a large increase in delayed imaging examinations from the pandemic.

During the pandemic, changes in radiology workflow were also witnessed. Prior to the pandemic, radiology was practiced either onsite through academic centers or group practice locations, as well as remotely though teleradiology services. With the need for social distancing during the pandemic, academic institutions often turned to virtual readouts to perform resident education [69]. Some institutions also permitted attending radiologists to read from home and perform readouts from outside the hospital [13]. The presence of virtual readouts and virtual rounding may be beneficial in providing flexibility to both the resident and attending radiologist, with residents often citing this as one of the largest benefits of remote learning [70]. In group practices, shift changes were seen to either increase the number of radiologists reading from home or by increasing a radiologist’s shift length to allow for increased staggering, leading to a decrease in the number of individuals onsite [71]. It is predicted such changes will persist beyond the current pandemic [72,73,74,75].

Imaging protocols were also implemented to limit both staff and patient exposure to potential COVID-19 positive individuals. In order to execute such protocols, remote webinar-based teachings were often held [76]. Such protocols involved the use of personal protective equipment (PPE) for both patients and staff, as well as cleaning and sanitation processes. Radiology technicians may also have had to don and doff in anterooms.

The use of portable imaging, such as portable x-ray imaging, for both COVID-19 suspected diagnoses and non-COVID-19 related diagnoses increased during the pandemic. Portable x-rays helped alleviate the need for CT imaging in the diagnosis of COVID-19 in many countries. However, they were also utilized for non-COVID-19 related studies. Studies also indicated that, despite the increase in portable x-rays, there was no significant increase in radiation exposure to the radiographers [28]. Furthermore, one institution studied the ability to perform a portable x-ray at over a distance of six feet through a glass door in order to preserve PPE materials for COVID-19-positive patients. The study found no significant decrease in image quality compared with the standard portable x-ray technique [77]. Similar techniques can be adapted when there is the need for sparing PPE or limited patient contact.

There are a few limitations to this review. While this review aims to be comprehensive, data regarding the pandemic’s effect on radiology operations have not been published internationally to the same extent as they have been domestically. Furthermore, only articles indexed in PubMed and Google Scholar were included in this study. This limits the total number of studies available and may limit the number of international manuscripts, as well as those published in languages other than English. This limits the scope of a global review. Additionally, data from private practice radiology groups were more limited and most data come from academic centers. Similarly, of the manuscripts reviewed, many did not contain quantitative data with regards to finances and volume. As a result, we were unable to perform a statistical analysis of the overall magnitude of volume and financial changes that occurred to radiology departments during the COVID-19 pandemic.

This review aimed to provide a global perspective on the effects of the COVID-19 pandemic on hospital systems and outpatient radiology practices. This review found that while the COVID-19 pandemic was handled differently in each country, the effects on radiology imaging volume were similar. Most practices and institutions noted a large decrease in overall imaging volume and, subsequently, revenue. Similarly, outpatient practices were more affected than hospital-based radiology departments as decreases were seen more largely in non-urgent or elective imaging procedures. During this period, many radiology centers had to determine how to best alter operations and workflow to ensure safety for both patients and staff. As regulatory restrictions ease, radiology departments will have to determine how to best rearrange staffing and operations to allow for the expected increase in imaging volumes.

## Data Availability

No new data was created for this study.
